# Understanding the complexity of socioeconomic disparities in type 2 diabetes risk: a study of 4.3 million people in Sweden

**DOI:** 10.1136/bmjdrc-2019-000749

**Published:** 2019-11-07

**Authors:** Maria Wemrell, Louise Bennet, Juan Merlo

**Affiliations:** 1 Unit for Social Epidemiology, Department of Clinical Sciences Malmö, Lund University, Malmö, Sweden; 2 Department of Gender Studies, Lund University, Lund, Sweden; 3 Unit for Family and Community Medicine, Department of Clinical Sciences Malmö, Lund University, Malmö, Sweden; 4 Center for Primary Health Care Research, Region Skåne, Malmö, Sweden

**Keywords:** type 2 diabetes, epidemiology, social determinants

## Abstract

**Objective:**

Investigating demographic and socioeconomic factors as intersecting rather than as separate dimensions may improve our understanding of the heterogeneous distribution of type 2 diabetes in the population. However, this complexity has scarcely been investigated and we still do not know the accuracy of these factors for predicting type 2 diabetes. Improved understanding of the demographic and socioeconomic disparities predicting type 2 diabetes risk in the population would contribute to more precise and effective public health interventions.

**Research design and methods:**

We analyzed the risk of type 2 diabetes among 4 334 030 individuals aged 40–84 years who by 2010 had resided in Sweden for at least 5 years. We stratified the study population into 120 strata defined by categories of age, gender, income, education, and immigration status. We calculated measures of absolute risk (prevalence) and relative risk (prevalence ratio), and quantified the discriminatory accuracy of the information for predicting type 2 diabetes in the population.

**Results:**

The distribution of type 2 diabetes risk in the population was highly heterogeneous. For instance, immigrated men aged 70–79 years with low educational achievement and low income had a risk around 32 times higher than native women aged 40–49 years with high income and high educational achievement (ie, 17.6% vs 0.5%). The discriminatory accuracy of the information was acceptable.

**Conclusion:**

A more detailed, intersectional mapping of socioeconomic and demographic distribution of type 2 diabetes can assist in public health management aiming to reduce the prevalence of the disease.

**Significance of this study**

**What is already known about this subject?**

- Socioeconomic status is an established determinant of type 2 diabetes.

**What are the new findings?**

- We investigate demographic and socioeconomic factors as intersecting rather than as separate dimensions, to improve our understanding of the distribution of type 2 diabetes in the population. In addition, we assess the discriminatory accuracy of these factors for predicting type 2 diabetes.

**How might these results change the focus of research or clinical practice?**

- Furthered understanding of the demographic and socioeconomic disparities predicting type 2 diabetes risk in the population can contribute to the precision and effectivity of public health interventions.

## Introduction

The global public health relevance of type 2 diabetes is today unquestionable. Type 2 diabetes is associated with a spectrum of macrovascular and microvascular complications contributing to morbidity and premature mortality. It has also been linked to cancer, depression, and infections.^1^ Overall, the prevalence of type 2 diabetes in Sweden in 2018 was approximated at 4%.^2^ However, due to sometimes unspecific symptoms at onset, which can be difficult for the individual to recognize, it has been estimated that about one-third of patients are unaware of their illness.^3^

A review of the literature^4^ has provided unquestionable evidence of socioeconomic status as a determinant of type 2 diabetes. It is well established that, in addition to genetic factors, the individual’s socioeconomic position in society contributes to the development of type 2 diabetes. Socioeconomic position conditions an array of both psychosocial^5^ and material^6^ factors like, for instance, psychosocial stress, access to healthcare services, availability of healthy foods and exercise, and individual lifestyle constraints or choices that affect the risk of developing type 2 diabetes.^7^

While type 2 diabetes is generally more prevalent among foreign-born than Swedish-born individuals,^8^ the prevalence varies considerably between different groups. It has been suggested that for Middle Eastern immigrants in Sweden, ethnicity itself should be considered a risk factor for type 2 diabetes, independent of anthropometric factors, heredity, lifestyle factors, and socioeconomic status.^9^ However, ethnic disparities in type 2 diabetes may largely be explained by socioeconomic variables.^10^ For example, long-term follow-up studies from Sweden have shown that refugees living in marginalized neighborhoods developed diabetes to a larger extent than those living in less deprived areas.^11^ Differences can further be seen between the genders. While men overall have a higher risk of type 2 diabetes than women,^12 13^ the opposite is true among people having immigrated to Sweden from areas such as Iraq, North Africa, South Asia, Syria, and Turkey.^14^ Moreover, the increased risk for type 2 diabetes among individuals with low socioeconomic status seems more pronounced among women than among men.^4 15 16^

Thus, the socioeconomic and demographic distribution of type 2 diabetes appears to be complex. For understanding such complexity, public health epidemiology is currently adopting an analytical framework inspired by intersectionality theory.^17–21^ Intersectionality theory posits that socioeconomic and demographic categories, such as socioeconomic status, sex/gender, and race/ethnicity or migration background, should be understood and analyzed not as separate but as interacting. The use of an intersectional framework represents a new way of understanding the complex nature of health inequities,^22^ and it may provide an improved awareness of the heterogeneous distribution of type 2 diabetes risk in the population.^17^ To the best of our knowledge, however, only one previous study from Canada has used an intersectional approach in the study of diabetes risk.^23^ Here, it should be emphasized that we are not referring to heterogeneity in terms of different diabetes phenotypes, but in terms of differences in the propensity for suffering from type 2 diabetes.

An improved understanding of the heterogeneous distribution of type 2 diabetes risk in the population would contribute to more precise public health interventions.^24 25^ In analogy with modern risk factor and biomarker research, the epidemiological and public health analysis of type 2 diabetes risk should be based on measures of average risk (eg, prevalence and relative risk) and also on assessments of discriminatory accuracy (DA), that is, the capacity of the risk factors at hand to correctly discriminate between people with or without the outcome of interest.^26 27^ In this study, we therefore aim to determine the accuracy of socioeconomic and demographic information for classifying individuals according to their type 2 diabetes status. We do so by analyzing a database that covers the total population of Sweden.

## Population and methods

### Databases

Our database was composed via record linkage between the Register of the Total Population (TPR) and the Longitudinal Integration Database for Health Insurance and Labour Market Studies (LISA)^28^ administered by Statistics Sweden, as well as the National Patient Register (NPR)^29^ and the Swedish Prescribed Drug Register (SPDR)^30^ administered by the National Board of Health and Welfare. The NPR records all hospital contacts including external visits to specialized care, while the SPDR records complete information on primary healthcare and covers all cases of dispensing from pharmacies except for hospital and nursing home storage. We performed the record linkage using the unique Personal Registration Number given to each individual residing in Sweden. However, Statistics Sweden and The National Board of Health and Welfare replaced these numbers with arbitrary serial numbers, for the purpose of anonymization, before delivering the files to the research group. The Regional Ethics Review Board in southern Sweden and the data safety committees from the National Board of Health and Welfare and from Statistics Sweden approved the construction of the database.

### Study population

According to the TPR, 9 415 488 individuals resided in Sweden at the baseline date of December 31, 2010. From this population, we excluded 4 864 402 individuals younger than 40 or older than 84 since type 2 diabetes is less frequent among those younger than 40 and the study of those older than 84 may require special considerations regarding the measurement of socioeconomic position, diseases, and use of medication. Thereafter, we excluded 96 130 individuals who had been living in Sweden for less than 5 years prior to the baseline date, and from whom we therefore have incomplete register information on socioeconomic information and previous diabetes. Subsequently, we excluded 68 016 individuals with a previous diagnosis (ICD-10 code) of type I diabetes mellitus (E10), malnutrition-related diabetes mellitus (E12), other specified diabetes mellitus (E13), or non-specified diabetes mellitus (E14). Lastly, we excluded 32 209 individuals with missing values on any of the socioeconomic or demographic variables.

**Figure 1:**
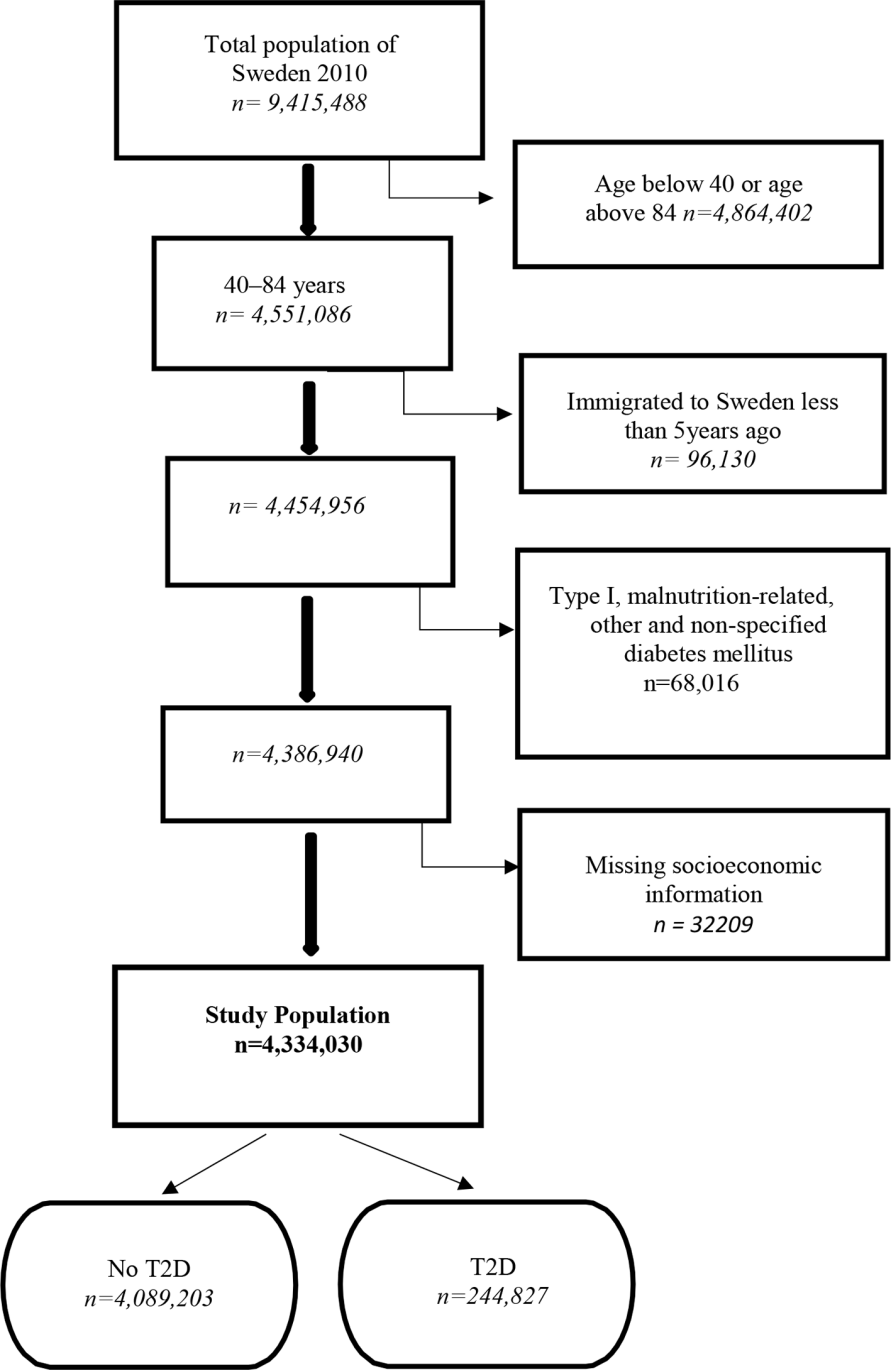
Flow chart documenting inclusion criteria, exclusion criteria, and the number of individuals included in the study population. T1D, type 1 diabetes; T2D, type 2 diabetes.

Finally, the database contained information on 4 334 030 individuals between 40 and 84 years of age, without any diabetes mellitus diagnosis other than type 2 diabetes, and with complete information regarding socioeconomic and demographic variables (figure 1).

### Assessment of variables

#### Type 2 diabetes

Individuals diagnosed with other types of diabetes (E10–14, see above) were already excluded from the study population. Therefore, we assumed that an individual suffered from type 2 diabetes if during the last 5 years he/she had (1) an ICD-10 code E11 or (2) at least one dispensation of oral antidiabetics coded as A10B according the Anatomical Therapeutic Chemical classification system, or (3) of insulins coded as A10A.

#### Demographic and socioeconomic variables

Gender was coded as male or female. Age at the baseline date was categorized into five groups by 10-year intervals, as 40–49, 50–59, 60–69, 70–79, and 80–84 years. Immigration status was classified into a binary variable, labeling individuals born in Sweden as natives, and individuals born in another country as immigrated.

In order to ensure an accurate measure of income, we based our variable on the individualized disposable income and computed a cumulative income for the years 2000, 2005, and 2010. We calculated this variable by dividing the total disposable income of the household with the number of individuals in that household, while simultaneously considering the different consumption weights of adults and children of different ages, according to criteria used by Statistics Sweden.^28^ Using the total Swedish population for each year (ie, not the study population), we computed 25 groups by quantiles in 2000, 2005, and 2010. Thereafter, we added the values from these 3 years and obtained a range of values from 3 (the lowest cumulative income) to 75 (the highest cumulative income). Finally, we categorized this cumulative income into three groups (high, middle, and low income) by dividing the range into tertiles.

We dichotomized the educational achievement variable into high or low educational attainment. To adjust for the rising level of education in Sweden, we categorized individuals younger than 65 as having high educational achievement if they had completed at least 2 years of university or equivalent, while classifying individuals above the age of 65 into the same category if they had attended at least 2 years of high school.

#### Multicategorical (intersectional) variable

In order to operationalize the intersectional approach discussed above, we created a multicategorical variable by combining the two categories of gender, the five categories of age, the two categories of immigration status, the three categories of cumulative income, and the two categories of educational achievement. This procedure created 120 intersectional strata (2×5×2 by 3×2), defined by the combined variables. We used native women 40–49 years old with high income and high educational achievement as our reference stratum, as this group was assumed, according to our available knowledge, to have the lowest type 2 diabetes risk.

### Statistical analyses

We performed seven consecutive regression analyses, modeling type 2 diabetes as the dependent variable. We applied logistic regression analysis as well as Cox proportional-hazards regression with a constant follow-up time equal to 1.^31^ Both kinds of analysis provided similar results. However, we show the results from the Cox regression as it gives prevalence ratios (PRs) rather than ORs. In all models, we calculated PRs with 99%, rather than 95%, CIs as we performed a large number of simultaneous analyses.

The first model adjusted for age only. Thereafter, we expanded model 1 by adding the single variables of gender (model 2), cumulative income (model 3), educational achievement (model 4), and immigration status (model 5). In model 6, we entered all five single variables simultaneously, and, finally, in model 7 we included our multicategorical (intersectional) variable.

For each model, we quantified its DA by means of the area under the receiver operator characteristic curve (AUC).^26^ The AUC is constructed by plotting the true-positive fraction (ie, sensitivity) against the false-positive fraction (ie, 1–specificity) for different binary classification thresholds of the predicted probability of type 2 diabetes. Thus, the AUC measures the accuracy of the information provided by the variables in the model for discriminating individuals with type 2 diabetes from those without it. The AUC takes a value between 0.5 and 1, where 1 indicates perfect discrimination and 0.5 means that the studied variables have no DA at all. There is no fully established practical guideline for the interpretation of the size of the AUC as a measure of DA when analyzing socioeconomic inequalities. However, based on the classification provided by Hosmer and Lemeshow,^32^ we defined the DA as “absent or very low” (AUC=0.5–0.6), “poor” (>0.6 to ≤0.7), “acceptable” (>0.7 to ≤0.8), “excellent” (>0.8 to ≤0.9), or “outstanding” (>0.9–1).

We further calculated the incremental change in the AUC value (ΔAUC) between the model including only age (model 1) and the respective consecutive models to quantify the value added by the chosen socioeconomic and demographic variables for classifying individuals according their type 2 diabetes status. While models 6 and 7 contain the same variables, the multicategorical variable used in model 7 allows for the capture of any interaction of effects. Therefore, the ΔAUC between models 6 and model 7 informs of the existence of any such interaction effect between the categories that define the strata, in relation to the reference stratum.^19^

We also performed graphical stratified analyses of absolute risk of type 2 diabetes and quantified income gradients in type 2 diabetes risk across strata defined by the other variables.

We used IBM SPSS V.22 for PC to perform all statistical analyses.

## Results

We identified 244 827 patients with type 2 diabetes among the 4 361 639 individuals aged 40–84 years, rendering an overall type 2 diabetes prevalence of 5.6%. However, as indicated in table 1, the prevalence was higher among men (6.7%) than among women (4.7%), while the risk increased with age to the extent that the prevalence was more than seven times higher in the oldest age group than in the youngest. As expected, we further found an income gradient in type 2 diabetes risk, meaning that the prevalence increased as the level of income decreased. Further, the type 2 diabetes risk was higher among immigrated people and among individuals with low educational achievement, compared with those born in Sweden and individuals with high educational achievement.

**Table 1:** Relative risk (prevalence ratio) of type 2 diabetes (T2D) in relation to age, gender, income, educational achievement, and immigration status as separate variables (models 1 to 6) and as a multicategorical variable (model 7) in the 2010 Swedish population aged 40 to 84 years

Individuals (% T2D)	Gender		Age					Income			Education		Immigration		Model 1	Model 2	Model 3	Model 4	Model 5	Model 6	Model 7
	Women	Men	40–49	50–59	60–69	70–79	80–84	High	Medium	Low	High	Low	Native	Immigrant	Prevalence ratio (99% CI)						
2 209 758 (4.7)																Ref				Ref	
2 124 272 (6.7)																1.51 (1.50 to 1.53)				1.57 (1.55 to 1.58)	
1 222 368 (1.5)															Ref	Ref	Ref	Ref	Ref	Ref	
1 110 124 (3.9)															2.55 (2.50 to 2.61)	2.56 (2.50 to 2.62)	2.67 (2.61 to 2.73)	2.52 (2.46 to 2.58)	2.56 (2.50 to 2.62)	2.61 (2.55 to 2.67)	
1 115 015 (7.6)															4.96 (4.86 to 5.06)	4.98 (4.88 to 5.09)	5.61 (5.49 to 5.72)	4.90 (4.80 to 5.00)	5.06 (4.96 to 5.17)	5.49 (5.38 to 5.61)	
654 694 (11.0)															7.16 (7.01 to 7.31)	7.28 (7.13 to 7.44)	7.30 (7.15 to 7.45)	6.93 (6.78 to 7.08)	7.31 (7.15 to 7.46)	7.37 (7.21 to 7.52)	
231 829 (11.4)															7.44 (7.26 to 7.63)	7.76 (7.57 to 7.95)	7.06 (6.89 to 7.24)	6.99 (6.82 to 7.16)	7.68 (7.49 to 7.87)	7.35 (7.17 to 7.53)	
1 535 656 (4.6)																	Ref			Ref	
1 348 687 (5.9)																	1.32 (1.30 to 1.34)			1.22 (1.21 to 1.24)	
1 449 687 (6.5)																	1.61 (1.59 to 1.63)			1.42 (1.40 to 1.44)	
1 442 573 (3.9)																		Ref		Ref	
2 891 457 (6.5)																		1.55 (1.53 to 1.57)		1.45 (1.43 to 1.47)	
3 736 190 (5.4)																			Ref	Ref	
587 840 (7.3)																			1.53 (1.51 to 1.55)	1.46 (1.44 to 1.48)	
The five multicategorical strata with the lowest prevalence of T2D																					
69 689 (0.5)																					Ref
87 561 (0.6)																					1.15 (0.97 to 1.37)
69 254 (0.7)																					1.24 (1.04 to 1.48)
52 388 (0.8)																					1.37 (1.13 to 1.64)
72 036 (0.8)																					1.39 (1.17 to 1.65)
The five multicategorical strata with the highest prevalence of T2D																					
7949 (15.1)																					27.56 (26.69 to 32.07)
3872 (16.1)																					29.45 (24.91 to 34.81)
16 960 (16.4)																					29.9 4 (26.01 to 34.45)
3170 (16.8)																					30.67 (25.81 to 36.46)
10 570 (17.6)																					32.10 (27.78 to 37.10)
AUC															0.684 (0.683 to 0.686)	0.698 (0.697 to 0.699)	0.698 (0.696 to 0.699)	0.695 (0.694 to 0.696)	0.692 (0.690 to 0.693)	0.717 (0.716 to 0.719)	0.720 (0.719 to 0.721)
ΔAUC																0.014	0.014	0.011	0.008	0.033	0.036

The colored boxes mark which categories the prevalence ratios concern, which in the multicategorical variable (model 7) are combinations of variables. Values are PR (prevalence ratios) with 99% CIs; AUC (area under the receiver operating characteristic curve) with 99% CI; ΔAUC (change in the AUC value compared with model 1).

Table 1 shows the five strata with the lowest and the highest type 2 diabetes prevalence, respectively (see information in online supplemental material S1). Unsurprisingly, the multicategorical stratum with the lowest type 2 diabetes risk (ie, only 0.5%) was that comprised by native women aged 40–49 years with high income and high educational achievement. In contrast, immigrated men aged 70–79 years with low educational achievement and low income had a type 2 diabetes risk of 17.6%, that is, about 32 times higher than the reference strata. The age-related increase in type 2 diabetes risk leveled out at around 70–80 years of age. Furthermore, the income gap clearly increased with age, especially among women.

10.1136/bmjdrc-2019-000749.supp1 Supplementary data

**Figure 2:**
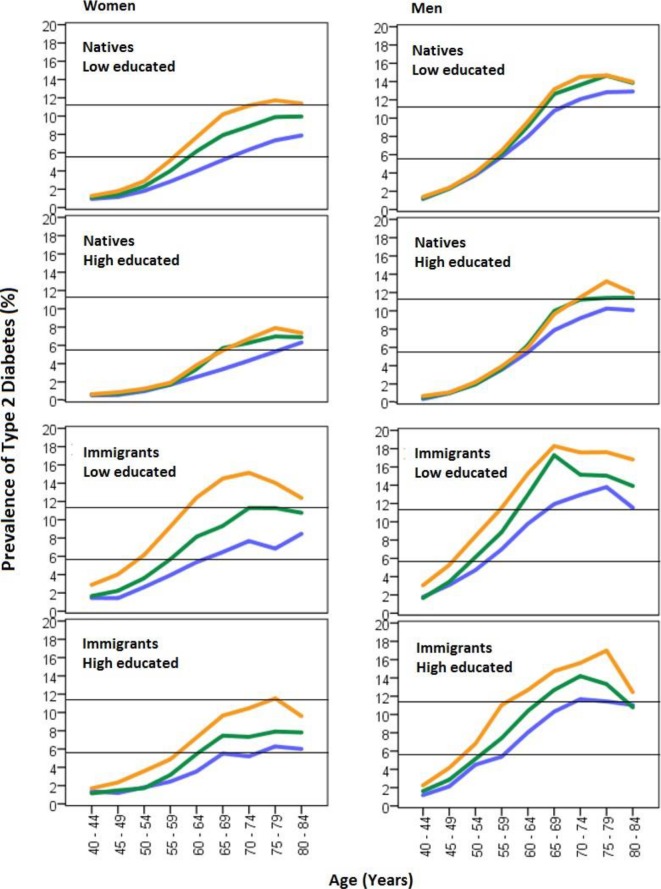
Prevalence (absolute risk) of type 2 diabetes by age, gender immigration status, educational achievement, and high (blue), medium (green), and low (orange) income levels.

When combining age, gender, educational achievement, and immigration status (figure 2), we could clearly observe that age accentuated the income gradient, that is, the differences in diabetes risk between low-income, middle-income, and high-income groups increased with age. This was especially evident among immigrated persons. The income gradient was also more emphasized among native women with low educational achievement, as compared with native women with high educational achievement. In relative terms (ie, PR), the income gradient in type 2 diabetes was steeper among women than among men (see online supplemental material S1).

**Figure 3:**
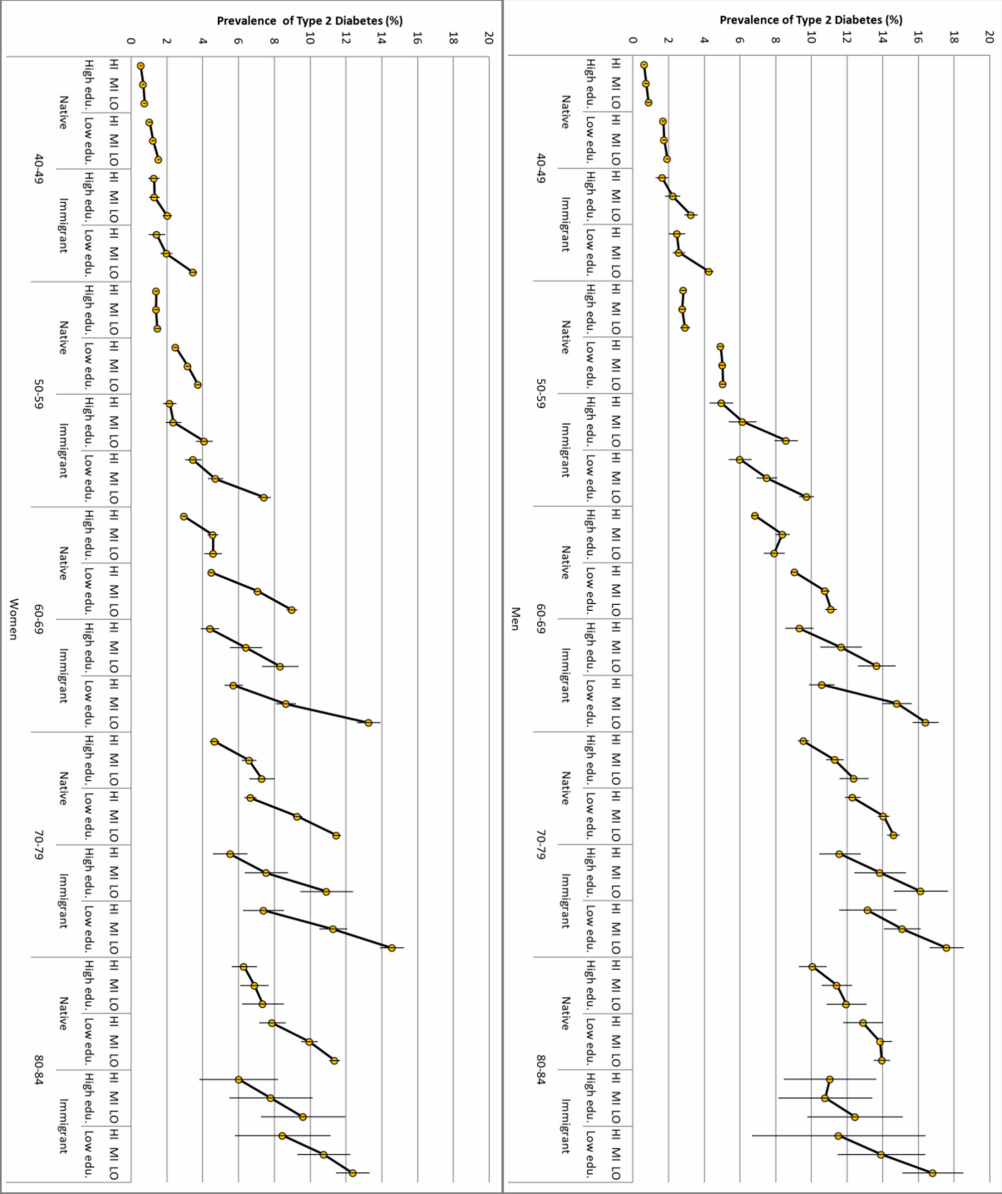
Prevalence (absolute risk) of type 2 diabetes (T2D) in men and women by multicategorical strata defined by age, gender, immigration status, educational achievement, and high (HI), medium (MI), and low (LO) income levels. The association between income and T2D risk is illustrated by thick black lines with yellow circles crossed by the lines representing 99% CIs.

Figure 3 shows substantial heterogeneity in distribution of risk for type 2 diabetes across the multicategorical/intersectional strata. The simple observation of the income gradients across strata shows very strong associations between decreasing income and increasing absolute risk for type 2 diabetes risk among people older than 60 years and, even more so, among immigrated individuals.

Table 1 shows the AUC values for the different models. The DA of age alone was *poor* (AUC=0.684). However, adding gender in model 2 (ΔAUC=0.014), income in model 3 (ΔAUC=0.014), educational achievement in model 4 (ΔAUC=0.011), or immigration status in model 5 (ΔAUC=0.008) only slightly improved the DA obtained by age alone. Entering all variables in model 6 slightly increased the DA (ΔAUC=0.033) to an *acceptable* value (AUC=0.717). The DA increased when the multicategorical variable was included in model 7, but it just remained acceptable (AUC=0.720). Hence, modeling the variables in the multicategorical form suggested the existence of interaction effects. However, the slight ΔAUC suggest that most of the observed risks were due to the additive effects of the demographic and socioeconomic variables defining the strata, while the interaction of effects was very small.

## Discussion

In alignment with previous research findings, this study confirms that the risk for type 2 diabetes increases with low socioeconomic position,^4^ immigration,^8^ male gender,^13 26^ and age. In addition, through adopting an intersectional perspective, we analyzed strata defined by combinations of those variables, which allowed us to reveal a large socioeconomic heterogeneity in type 2 diabetes.^4^

We were able to identify strata with very high risk for type 2 diabetes, such as elderly immigrated men with low income and low educational achievement, and strata with very low type 2 diabetes risk, like native women 40–49 years old with high income and high educational achievement. Furthermore, we found that the income gradient clearly became more pronounced with age, suggesting that the negative biological and social consequences of a low income can accumulate across the life course and contribute to a higher type 2 diabetes risk later in life. Figure 2 indicates that the age-related increase in type 2 diabetes risk leveled out at around 70–80 years of age. However, this pattern could be explained by a slightly lower life expectancy among patients with type 2 diabetes, compared with non-diabetics.

It is noteworthy that, in relative terms, income gradients in type 2 diabetes risk were more pronounced among women than among men, and among immigrated individuals compared with those born in Sweden. Among women with low educational attainment, income also had a larger impact on type 2 diabetes risk than among highly educated women. The steeper income gradient among women may—at least in part—be explained by the lower absolute risk among women overall. It has further been shown that among women, but not among men, most socioeconomic differences in excess risk of type 2 diabetes can be explained by established risk factors such as overweight, physical inactivity, smoking, and heredity in combination with psychosocial factors such as low decision latitude at work and a low sense of coherence.^33^

Concerning the higher overall prevalence ratio for type 2 diabetes among immigrated persons, this may be partially due to genetic and lifestyle factors.^8^ While ethnic background seems to play a role in the risk for type 2 diabetes,^34^ the DA of this variable beyond age, gender, and socioeconomic position has—to the best of our knowledge—still not been quantified. Immigration status represents a very vague proxy for ethnicity and, as such, does not properly capture any possible biological effects pertaining to ethnicity on risk for type 2 diabetes. However, while the immigrant group is internally very heterogeneous ethnically, the burden of a low income may particularly affect immigrated persons, irrespective of ethnic diversity. For instance, the absolute risk of type 2 diabetes among women with high income and high education was similar among natives and immigrants, while the difference in absolute risk in the low-income group was larger among immigrated persons. Immigrated persons, particularly refugees, are further commonly uniquely exposed to stressful events and unhealthy life circumstances and lifestyles due to, for example, involuntary displacement, experiences of discrimination, and habitation in socioeconomically vulnerable neighborhoods.^11^ In any case, the DA of this variable was found to be very low.

The analysis of DA indicated that the combined information on age, gender, income, education, and immigration status gave an *acceptable* DA (AUC=0.720) with regards to type 2 diabetes. This fact, combined with the high absolute risk for type 2 diabetes in some intersectional strata, provides relevant information for the planning of more “precise” public health interventions. The framework we propose also fits well with the idea of proportionate universalism discussed by Marmot and Bell with regards to resource allocation in public health.^35 36^ That is to say, public health and preventive medicine actions must be universal, not targeted, but with a scale and intensity that is proportionate to the level of disadvantage of the recipients. Targeted intervention to population groups with a high risk for type 2 diabetes seems more appropriate when DA is acceptable, as in our case.

Intersectionality theory points toward experienced interactions between demographic and socioeconomic dimensions, meaning that the term interaction here reaches well beyond the statistical concept of interaction effects, as distinguished from additive or main effects, used by epidemiologists.^17^ Still, our study indicates the existence of additive effects between the socioeconomic and demographical variables used to define the intersectional strata. Interaction of effects was also present, but they were of minor relevance for understanding the risk of type 2 diabetes in the Swedish population.

### Strengths and limitations

From the perspective of intersectionality theory, this study can be perceived as consisting of a relatively simplistic treatment of the complex social structures that condition the distribution of power, resources, and health in society.^21^ In addition, intersectionality theory posits that intersectional identities cannot be decomposed as is done in our statistical analyses. However, the primary focus of our stratified analysis was to provide an improved mapping of the demographic and socioeconomic heterogeneity of type 2 diabetes. For this purpose, we drew on intersectionality theory to improve our analysis of socioeconomic health disparities.

A major strength of the study is that it is based on the analysis of a database containing the total Swedish population, which enabled extended and precise stratified analysis. While our definition of type 2 diabetes deviates somewhat from that used in the national diabetes register (NPR),^37^ it rests on register information about hospital diagnoses (both inpatients and external visits) as well as on specific medication for type 2 diabetes. Moreover, patients with type 1 diabetes were excluded, which may ensure the use of insulin as a proxy for type 2 diabetes. However, patients with other types of diabetes mellitus (such as latent autoimmune diabetes in adults) might have been misdiagnosed with type 2 diabetes, and vice versa. This could also be the case with a number of patients with MODY (Maturity Onset Diabetes in Young) since by 2010 this type of diabetes was still not coded in the NPR. A few patients in our study population might also have used metformin for polycystic ovary syndrome. In addition, in a nationwide population study, underdiagnosis of type 2 diabetes is inevitable since many individuals are unaware of their disease. However, since we included all patients on antidiabetic pharmacological treatment at any point from 2006 to 2010, we still included a major share of all patients with type 2 diabetes. We have not performed a formal validation study, but we think the validity of the type 2 diabetes diagnosis is not a major concern for our study. An alternative approach could be to identify cases of type 2 diabetes from the NPR.^38^ However, it could be possible that undiagnosed type 2 diabetes is more frequent in deprived groups and therefore our results may underestimate the existing socioeconomic differences.

The aim of this article was to document the heterogeneity of the demographic and socioeconomic distribution of type 2 diabetes in Sweden, rather than to build a predictive model that can be used in other contexts. However, the results are very similar when using bootstrapping for internal validation according to current recommendations.^39^ In any case, on the basis of this study, we can draw conclusions about correlations, but not about causal relationships.

Socioeconomic factors are conceptualized as root or upstream determinants of disease. That is to say, from a longitudinal perspective, many diseases are mediators rather than independent causes. Moreover, we did not consider the influence of the neighborhood environment in our analyses. Hence, extended analyses including certain diseases and/or neighborhood environmental factors may discover further heterogeneity.

It should further be noted that while a previously more compressed distribution of income in Sweden has in recent years given way to an increasing economic inequality,^40^ formerly relatively narrow income gaps in combination with remaining tenets of a strong social welfare system including universal healthcare may limit the generalizability of our findings, by attenuating the relationship between the socioeconomic variables and type 2 diabetes risk.

## Conclusion

Through studying several socioeconomic and demographic variables in combination, our study revealed a complex heterogeneity of type 2 diabetes risk. This mapping of type 2 diabetes risk, drawing on intersectionality theory, represents a contribution to previous research in the field.^4^ The analysis indicated that the combined information about age, gender, income, education, and immigration status gave an acceptable DA. This fact, together with the large absolute risk for type 2 diabetes in some intersectional strata, provides important information for more precise public health interventions.^24 25 41^ Consideration of group average differences in type 2 diabetes risk as well as the DA of this information represents an improved analytical strategy, in correspondence with the principles of proportionate universalism,^35 36^ for informing decisions regarding the degree to which public health interventions need to be universal or targeted.
