# Supplementary material for: Understanding the complexity of socioeconomic disparities in type 2 diabetes risk: a study of 4.3 million people in Sweden
Source: BMJ Open Diabetes Res Care. 2019 Nov 7;7(1):e000749. doi: 10.1136/bmjdrc-2019-000749 (PMC6861116; doi:10.1136/bmjdrc-2019-000749)
Supplement: Supplementary data [file bmjdrc-2019-000749supp001.pdf]

Supplemental information, S1

Number, prevalence and prevalence ratio of type 2 diabetes among multicategorical strata. CI: Confidence Intervals.

Gender	Age	Immigration status	Educational achievement	Income	Number	Prevalence by percentage (99% CI)	Prevalence Ratio (99% CI)
Women	40 – 49	Native	High	High	69 689	0.6 (0.5–0.6)	Ref
				Middle	69 254	0.7 (0.6–0.8)	1.2 (1.0–1.5)
				Low	72 036	0.8 (0.7–0.8)	1.4 (1.2–1.6)
			Low	High	44 580	1.0 (0.9–1.2)	1.9 (1.6–2.3)
				Middle	84 069	1.2 (1.1–1.3)	2.2 (1.9–2.6)
				Low	163 010	1.5 (1.5–1.6)	2.8 (2.4-3.2)
		Immigrant	High	High	8 175	1.3 (1.0–1.6)	2.3 (1.8–3.1)
				Middle	9 722	1.3 (1.0–1.6)	2.4 (1.8–3.1)
				Low	19 426	2.0 (1.8–2.3)	3.7 (3.1–4.4)
			Low	High	4 389	1.4 (1.0–1.9)	2.6 (1.8–3.7)
				Middle	11 077	2.0 (1.6–2.3)	3.6 (2.9–4.5)
				Low	48 035	3.5 (3.3–3.7)	6.3 (5.5–7.3)
	50–59	Native	High	High	80 362	1.4 (1.3–1.5)	2.6 (2.2–3.0)
				Middle	56 068	1.4 (1.3–1.5)	2.6 (2.2–3.0)
				Low	39 016	1.5 (1.3–1.6)	2.7 (2.3–3.2)
			Low	High	89 147	2.5 (2.4–2.6)	4.5 (3.9–5.2)
				Middle	101 737	3.2 (3.0–3.3)	5.8 (5.0–6.6)
				Low	97 453	3.8 (3.6–3.9)	6.8 (6.0–7.9)
		Immigrant	High	High	9 320	2.2 (1.8–2.5)	3.9 (3.1–4.9)
				Middle	8 279	2.4 (1.9–2.8)	4.3 (3.4–5.4)
				Low	11 673	4.1 (3.6–4.6)	7.5 (6.3–8.9)
			Low	High	10 375	3.5 (3.0–3.9)	6.3 (5.2–7.6)
				Middle	16 717	4.7 (4.3–5.1)	8.6 (7.3–10.1)
				Low	33 990	7.4 (7.1–7.8)	13.6 (11.8–15.6)
	60 – 69	Native	High	High	120 862	3.0 (2,8–3,1)	5.4 (4.7–6.2)
				Middle	32 242	4.6 (4.3–4.9)	8.3 (7.2–9.7)
				Low	12 461	4.6 (4.1–5.1)	8.4 (7.1–9.9)
			Low	High	146 071	4.5 (4.4–4.6)	8.2 (7.1–9.4)
				Middle	120 775	7.1 (6.9–7.3)	12.9 (11.3–14.8)
				Low	63 247	9.0 (8.7–9.3)	16.4 (14.3–18.8)
		Immigrant	High	High	10 396	4.4 (3.9–4.9)	8.1 (6.7–9.6)
				Middle	4 995	6.4 (5.5–7.3)	11.7 (9.6–14.2)
				Low	4 801	8.3 (7.3–9.4)	15.2 (12.6–18.3)
			Low	High	13 451	5.7 (5.2–6.2)	10.4 (8.9–12.3)
				Middle	16 032	8.7 (8.1–9.2)	15.8 (13.6–18.3)
				Low	18 315	13.3 (12.6–13.9)	24.2 (21.0–27.9)
	70 – 79	Native	High	High	40 353	4.7 (4.4–4.9)	8.5 (7.4–9.8)
				Middle	24 277	6.6 (6.2–7.0)	12.0 (10.4–13.9)
				Low	8 893	7.3 (6.6–8.0)	13.3 (11.3–15.7)
			Low	High	37 792	6.7 (6.3–7.0)	12.2 (10.6-14.0)

Gender	Age	Immigration status	Educational achievement	Income	Number	Prevalence by percentage (99% CI)	Prevalence Ratio (99% CI)
Women				Middle	85 740	9.3 (9.0–9,6)	17.0 (14.8–19.4)
				Low	111 084	11.5 (11.2–11.7)	20.9 (18.3–23.9)
		Immigrant	High	High	3 683	5.5 (4.6–6.5)	10.1 (8.1–12.6)
				Middle	3 152	7.6 (6.3–8.8)	13.8 (11.1–17.0)
				Low	2 995	10.9 (9.5–12.4)	19.9 (16.4–24.2)
			Low	High	3 438	7.4 (6.2–8.5)	13.5 (10.9–16.6)
				Middle	10 889	11.3 (10.5–12.1)	20.6 (17.7–23.9)
				Low	18 757	14.6 (13.9–15.2)	26.6 (23.1–30.6)
	80 – 84	Native	High	High	7 886	6.3 (5.6–7.0)	11.5 (9.7–13.7)
				Middle	6 997	6.9 (6.1–7.7)	12.6 (10.5–15.0)
				Low	3 344	7.4 (6.2–8.5)	13.4 (10.9–16.6)
			Low	High	8 792	7.9 (7.1–8.6)	14.4 (12.2–16.9)
				Middle	27 425	10.0 (9.5–10.4)	18.2 (15.8–20.9)
				Low	68 403	11.4 (11.1–11.7)	20.7 (18.1–23.7)
		Immigrant	High	High	782	6.0 (3.8–8.2)	11.0 (7.4–16.3)
				Middle	883	7.8 (5.5–10.1)	14.3 (10.2–20.0)
				Low	1 031	9.6 (7.2–12.0)	17.5 (13.1–23.4)
			Low	High	721	8.5 (5.8–11.1)	15.4 (10.8–22.0)
				Middle	2 936	10.8 (9.3–12.2)	19.6 (16.1–23.9)
				Low	8 258	12.4 (11.5–13.3)	22.6 (19.4–26.4)
Men	40 – 49	Native	High	High	87 561	0.6 (0.6–0.7)	1.2 (1.0–1.4)
				Middle	52 388	0.8 (0.7–0.9)	1.4 (1.1–1.6)
				Low	32 939	0.9 (0.8–1.0)	1.6 (1.3–2.0)
			Low	High	100 775	1.7 (1.6–1.8)	3.1 (2.7–3.6)
				Middle	115130	1.8 (1.7–1.9)	3.2 (2.8–3.7)
				Low	135 850	1.9 (1.8–2.0)	3.5 (3.1–4.1)
		Immigrant	High	High	8 095	1.6 (1.3–2.0)	3.0 (2.3–3.9)
				Middle	8 094	2.2 (1.8–2.7)	4.1 (3.2–5.1)
				Low	15 323	3.3 (2.9–3.6)	5.9 (5.0–7.1)
			Low	High	7 457	2.5 (2.0–2.9)	4.5 (3.6–5.7)
				Middle	13 746	2.6 (2.2–2.9)	4.7 (3.9–5.7)
				Low	41 548	4.3 (4.0–4.5)	7.8 (6.7–9.0)
	50 – 59	Native	High	High	75 944	2.8 (2.7–3.0)	5.2 (4.5–6.0)
				Middle	41 746	2.8 (2.6–3.0)	5.0 (4.3–5.9)
				Low	25 374	2.9 (2.7–3.2)	5.3 (4.5–6.3)
			Low	High	111 734	4.9 (4.8–5.1)	9.0 (7.8–10.3)
				Middle	110 392	5.0 (4.9–5.2)	9.2 (8.0–10.5)
				Low	108 341	5.1 (4.9–5.2)	9.2 (8.0–10.6)
		Immigrant	High	High	6 781	5.0 (4.3–5.6)	9.0 (7.5–11.0)
				Middle	6 365	6.2 (5.4–6.9)	11.2 (9.3–13.5)
				Low	12 481	8.6 (7.9–9.2)	15.7 (13.4–18.3)
			Low	High	9 270	6.0 (5.4–6.6)	11.0 (9.2–13.0)
				Middle	13 683	7.5 (6.9–8.1)	13.7 (11.7–16.0)
				Low	33 876	9.7 (9.3–10.2)	17.8 (15.5–20.4)

Gender	Age	Immigration status	Educational achievement	Income	Number	Prevalence by percentage (99% CI)	Prevalence Ratio (99% CI)
Men	60 – 69	Native	High	High	125 187	6.9 (6.7–7.0)	12.5 (10.9–14.3)
				Middle	32 254	8.4 (8.0–8.8)	15.3 (13.3–17.6)
				Low	14 114	7.9 (7.3–8.5)	14.5 (12.4–16.8)
			Low	High	153 698	9.1 (8.9–9.3)	16.5 (14.5–18.9)
				Middle	102 738	10.8 (10.5–11.0)	19.6 (17.2–22.5)
				Low	60 890	11.1 (10.8–11.4)	20.3 (17.7–23.2)
		Immigrant	High	High	8 919	9.3 (8.5–10.1)	17.0 (14.5–20.0)
				Middle	5 039	11.7 (10.5–12.8)	21.3 (18.0–25.2)
				Low	6 936	13.7 (12.6–14.7)	24.9 (21.3–29.1)
			Low	High	12 187	10.6 (9.9–11.3)	19.3 (16.6–22.4)
				Middle	12 445	14.8 (14.0–15.6)	27.0 (23.4–31.2)
				Low	16 960	16.4 (15.7–17.1)	29.9 (26.0–34.5)
	70 – 79	Native	High	High	54 881	9.6 (9.2–9.9)	17.4 (15.2–20.0)
				Middle	27 851	11.3 (10.8–11.8)	20.6 (18.0–23.7)
				Low	10 571	12.4 (11.6–13.2)	22.6 (19.5–26.3)
			Low	High	37 590	12.3 (11.9–12.8)	22.5 (19.6–25.8)
				Middle	72 411	14.1 (13.7–14.4)	25.6 (22.4–29.3)
				Low	66 079	14.6 (14.3–15.0)	26.6 (23.3–30.5)
		Immigrant	High	High	5 080	11.6 (10.4–12.8)	21.2 (17.9–25.1)
				Middle	3 847	13.9 (12.4–15.3)	25.3 (21.3–30.0)
				Low	3 872	16.1 (14.6–17.7)	29.4 (24.9–34.8)
			Low	High	2 940	13.2 (11.6–14.8)	24.0 (19.9–28.9)
				Middle	7 949	15.1 (14.1–16.1)	27.6 (23.7–32.1)
				Low	10 570	17.6 (16.6–18.6)	32.1 (27.8–37.1)
	80 – 84	Native	High	High	10 121	10.1 (9.3–10.8)	18.4 (15.7–21.4)
				Middle	9 291	11.4 (10.6–12.3)	20.9 (17.9–24.3)
				Low	5 567	12.0 (10.8–13.1)	21.8 (18.5–25.7)
			Low	High	5 917	12.9 (11.8–14.0)	23.6 (20.0–27.7)
				Middle	17 805	13.9 (13.2–14.5)	25.3 (21.9–29.1)
				Low	37 986	14.0 (13.5–14.4)	25.5 (22.2–29.2)
		Immigrant	High	High	969	11.0 (8.5–13.6)	20.1 (15.2–26.7)
				Middle	918	10.8 (8.2–13.4)	19.7 (14.7–26.3)
				Low	1 012	12.5 (9.8–15.1)	22.7 (17.4–29.6)
			Low	High	286	11.5 (6.7–16.4)	21.0 (13.2–33.6)
				Middle	1 329	13.9 (11.5–16.4)	25.4 (20.2–32-0)
				Low	3 170	16.8 (15.1–18.5)	30.7 (25.8–36.5)
